# Modulatory Effects of the Recombinant Middle East Respiratory Syndrome Coronavirus (MERS-CoV) Spike S1 Subunit Protein on the Phenotype of Camel Monocyte-Derived Macrophages

**DOI:** 10.3390/biology14030292

**Published:** 2025-03-13

**Authors:** Jamal Hussen, Abdullah I. A. Al-Mubarak, Turke Shawaf, Khulud Bukhari, Khaled R. Alkharsah

**Affiliations:** 1 Department of Microbiology, College of Veterinary Medicine, King Faisal University, Al-Ahsa 31982, Saudi Arabia; aiamubarak@kfu.edu (A.I.A.A.-M.); kbukh@kfu.edu.sa (K.B.); 2 Department of Clinical Sciences, College of Veterinary Medicine, King Faisal University, Al-Ahsa 31982, Saudi Arabia; tshawaf@kfu.edu.sa; 3 Department of Microbiology, College of Medicine, Imam Abdulrahman Bin Faisal University (IAU), Dammam 34212, Saudi Arabia; kalkharsah@iau.edu.sa

**Keywords:** MERS-CoV, spike protein, DPP4, macrophages, camel

## Abstract

Middle East Respiratory Syndrome Coronavirus (MERS-CoV) is an emerging zoonotic pathogen with different pathogenesis in humans and camels. The mechanisms behind the higher tolerance of camels to MERS-CoV infection are still unknown. Monocytes are innate myeloid cells that are able, depending on the local stimulation in their microenvironment, to differentiate into different functional subtypes of macrophages with an impact on the adaptive immune response. Several in vitro protocols have been used to induce the differentiation of monocyte-derived macrophages (MDMs) in human and several veterinary species. Such protocols are not available for camel species. In the present study, monocytes were separated from camel blood and differentiated in vitro in the presence of different stimuli into MDM. Camel MDMs generated in the presence of a combined stimulation of monocytes with LPS and GM-CSF resulted in the development of an M1 macrophages phenotype with increased abundance of the antigen-presentation receptor MHCII molecules and a decreased expression of the scavenger receptor CD163. The expression pattern of the cell markers CD163, CD14, CD172a, CD44, and CD9 on MDM generated in the presence of the MERS-CoV S1 protein revealed similarity with M-CSF-induced MDM, suggesting the potential of the MERS-CoV S1 protein to induce an M2 macrophages phenotype. Similarly to the effect of M-CSF, MERS-CoV-S protein-induced MDMs showed enhanced phagocytosis activity compared to non-polarized or LPS/GM-CSF-polarized MDMs. Collectively, our study represents the first report on the in vitro generation of monocyte-derived macrophages (MDMs) in camels and the characterization of some phenotypic and functional properties of camel MDM under the effect of M1 and M2 polarizing stimuli. In addition, the results suggest a polarizing effect of the MERS-CoV S1 protein on camel MDMs, developing an M2-like phenotype with enhanced phagocytosis activity. To understand the clinical relevance of these in vitro findings on disease pathogenesis and camel immune response toward MERS-CoV infection, further studies are required.

## Simple Summary

The current study represents the first report on the in vitro generation and polarization of monocyte-derived macrophages (MDMs) in camels and the impact of the MERS-CoV S1 protein on camel MDM phenotype. The results show a polarizing effect of the MERS-CoV S1 protein on camel MDM, turning it into an anti-inflammatory M2-like phenotype with enhanced phagocytosis activity.

## 1. Introduction

Middle East Respiratory Syndrome Coronavirus (MERS-CoV) is an emerging zoonotic pathogen responsible for the Middle East Respiratory Syndrome (MERS) [1,2,3,4,5]. Dromedary camels are considered to be the main source of zoonotic MERS-CoV infection in humans [6,7,8,9,10,11,12,13]. Recent serologic studies revealed very high seroprevalence rates of MERS-CoV in camel populations from different areas of Africa and the Arabian Peninsula [14]. MERS-CoV infection, however, shows different pathogenesis in humans and camels. Infection in humans leads to severe respiratory disease usually associated with hospitalization or death, while infected camels show only mild respiratory symptoms [15].

The type II transmembrane glycoprotein CD26, also called dipeptidyl peptidase 4 (DPP4), is the functional receptor for MERS-CoV [16,17,18]. Different anatomical expressions of DPP4 have been found in the respiratory tracts of humans and camels [19]. In camels, DPP4 was only found on epithelial cells of the upper respiratory tract, while, in humans, it is expressed on the epithelium of the lower respiratory tract [19]. In addition, dromedary camels display the highest expression of DPP4 on blood monocytes [20,21], contrary to humans, where DPP4 is mainly found on lymphocytes [22]. Whether this different distribution of the DPP4 on immune cells reflects different roles for innate and adaptive immune mechanisms in MERS-CoV pathogenesis in the two species is still to be investigated.

Monocytes are short-lived innate myeloid cells that circulate in the blood and contribute to successful immune response through several effector functions [23,24]. In addition, monocytes are responsible for the replenishment of long-lived tissue macrophages upon leaving the bloodstream and migrating into the tissue [25]. Depending on local stimulation in their microenvironment, human and murine monocytes can differentiate into different functional subtypes of macrophages with different roles during the onset and resolution of the immune response [26,27,28,29,30,31,32,33,34,35]. The granulocyte–macrophage colony-stimulating factor (GM-CSF), which is a macrophage maturation-promoting cytokine especially produced by inflammatory T cells (Th1 and Th17) under inflammatory conditions, induces, in combination with pathogen-associated molecular patterns like bacterial lipopolysaccharide (LPS), the generation of classically activated inflammatory (M1) macrophages [36,37]. In contrast, the generation of alternatively activated anti-inflammatory (M2) macrophages can be induced by the macrophage colony-stimulating factor (M-CSF) together with the type 2 cytokine IL-4 [36,37]. Inflammatory M1 macrophages play a significant role in the early control of infection through the production of inflammatory mediators, including the inflammatory cytokines IL-12 and TNFa and the enzyme iNOS, which catalyzes the production of nitric oxide (NO) from L-arginine. On the other hand, M2 macrophages are mainly important during the late resolution phase of the infection, where they predominantly produce higher levels of anti-inflammatory cytokines such as IL-10 and anti-inflammatory enzymes like arginase-1, which mediates the conversion of arginine to ornithine [38,39].

The response of human monocyte-derived macrophages toward experimental in vitro infection with MERS-CoV and SARS-CoV was comparatively investigated [40]. Although only MERS-CoV was able to infect the cells, both viruses shared some similarity in the cytokine response, with comparable levels of TNF-a and interleukin (IL)-6, but lacked type 1 interferon response (IFN-α and IFN-β). The notably high expression levels of type 1 cytokines IL-12 and IFN-γ, together with the high expression of major histocompatibility complex (MHC) molecules and costimulatory molecules, indicate the higher potential of MERS-CoV to stimulate classically activated inflammatory M1 macrophages compared to SARS-CoV. The inflammatory nature of these cells is also confirmed by the high levels of monocyte and neutrophil chemoattractants (CXCL-10, CCL-2, CCL-3, CCL-5, and IL-8) [40]. Additionally, the potential of SARS-CoV-2 S-polarized human monocytes and macrophages to induce the differentiation of helper T cells toward the Th17 phenotype has been recently reported [41]. In camels, studies on the interaction of MERS-CoV with innate immune cells are still lacking. It is especially interesting to see whether the interaction of camel monocytes (express CD26) with the MERS-CoV S protein would impact their differentiation into polarized macrophages.

Although the immunophenotype of camel blood monocytes has been recently characterized [42], their in vitro differentiation into monocyte-derived macrophages (MDMs) and their polarization into distinct subtypes of camel MDM under the effect of different stimuli have not been investigated so far. Therefore, the present study aimed to analyze some phenotypic and functional properties of in vitro-generated and -polarized camel MDM. Additionally, the impact of the MERS-CoV S protein on the in vitro differentiation of camel MDM was investigated.

## 2. Materials and Methods

### 2.1. Animals and Sampling

Blood samples were collected from five healthy female dromedary camels of the Mojaheem breed (Camel Research Center of King Faisal University, Al-Ahsa, Saudi Arabia). Blood samples were drawn into heparinized vacutainer tubes (Becton Dickinson, Heidelberg, Germany) from the jugular vein. The samples were kept on ice and delivered to the lab within one hour of collection. Cell separation from the collected blood samples was performed within two hours after sampling.

### 2.2. Reagents

Lipopolysaccharide (LPS) from *E. coli* serotype 0111:B4 (tlrl-eblps) was from Invivogen (Toulouse, France), diluted in endotoxin-free water to a stock concentration of 1 mg/mL, and stored in small aliquots at −20 °C. Recombinant bovine granulocyte–macrophage colony-stimulating factor (GM-CSF) and macrophage colony-stimulating factor (M-CSF) were from Kingfisher (Kingfisher Biotech, Inc., St Paul, MN, USA). Recombinant MERS-CoV (Middle East Respiratory Syndrome Coronavirus) Spike S1 Subunit Fc chimera protein was from R&D Systems, Inc. (Catalog Number: 10606-CV; Abingdon, UK). Human IgG conjugated with FITC was from Sigma (Sigma-Aldrich, St. Louis, MO, USA). The lymphocyte separation medium Lymphoprep™ was from STEMCELL Technologies (Vancouver, BC, Canada). The mouse monoclonal antibodies to CD14 (clone Tuk4), MHCII (clone TH81A5), CD163 (clone LND68A), CD172a (DH59b), and CD44 (clone LT41A) were from Kingfisher (Kingfisher Biotech, Inc., USA). Fluorescein isothiocyanate (FITC)- and phycoerythrin (PE)-conjugated goat antibodies to mouse IgG1 and IgG2a were from Thermo Fisher Scientific (Thermo Fisher Scientific, Waltham, MA, USA).

### 2.3. Isolation of Peripheral Blood Mononuclear Cells (PBMCs) from Camel Blood

Peripheral blood mononuclear cells (PBMCs) from dromedary camels were isolated from buffy coat blood by density gradient centrifugation over Lymphoprep™ (STEMCELL Technologies, Vancouver, BC, Canada). Collected blood (20 mL) was diluted with 15 mL of phosphate-buffered saline (PBS), and the mixture was layered carefully (without mixing the blood and the Lymphoprep) on 15 mL of Lymphoprep™ in a 50 mL sterile falcon tube. The tubes were then centrifuged at 4 °C for 30 min at 800× *g* without break. After centrifugation, the PBMC-containing interphase was collected carefully using a 10 mL pipette, washed 3 times in PBS (400× *g*, 200× *g*, 100× *g*), and finally suspended in culture medium [43].

### 2.4. Binding of the Recombinant MERS-CoV S1 Protein on the Cell Surface of Camel Mononuclear Cells

The binding of the recombinant MERS-CoV S1 protein on the cell surface of camel mononuclear cells was evaluated by flow cytometry as previously described, with modifications [21,44]. Isolated camel PBMCs (1 × 10^6^) in 100 µL cell-staining buffer (PBS containing 1% BSA and 0.1% sodium azide) were incubated with the recombinant MERS-CoV spike S1 subunit Fc chimera protein (1 µg/mL) for 30 min at 4 °C. After washing the cells with 150 µL of cell-staining buffer (300× *g* for 3 min at 4 °C), a polyclonal rabbit anti-human IgG labeled with FITC (DakoCytomation, Copenhagen, Denmark; Ref: F0202; diluted 1:200 in cell-staining buffer) was added to the cells for a further 15 min at 4 °C in the dark. To exclude the role of FC binding to FC receptors, a separate setup using human IgG conjugated with FITC was included. Finally, the cells were washed twice with 150 µL of cell-staining buffer (300× *g* for 3 min at 4 °C), resuspended in cell-staining buffer containing propidium iodide (PI), and analyzed by flow cytometry (Accuri C6 flow cytometer; BD Biosciences, Franklin Lakes, NJ, USA).

### 2.5. Monocyte Isolation and In Vitro Differentiation into Macrophages

Camel monocytes were separated using plastic adhesion as previously described, with some modifications [45]. Separated camel PBMCs were allowed to adhere in 24-well culture plates (Nunc, Rochester, NY, USA) for 24 h at 37 °C and 5% CO_2_ in serum-free culture media (RPMI 1640, supplemented with 2mM L-glutamine and 5 mM HEPES, MOLEQULE-ON, New Lynn, Auckland, New Zealand). After incubation, the non-adherent lymphocytes were removed by washing with a fresh culture medium. Adherent monocytes were thereafter differentiated in the same plates for 5 days at 37 °C and 5% CO_2_ in RPMI 1640 supplemented with 2 mM L-glutamine, 5 mM HEPES, and 10% inactivated fetal bovine serum (all from MOLEQULE-ON, New Lynn, Auckland, New Zealand). For the generation of classically activated M1 monocyte-derived macrophages (MDMs), 1 µg/mL of LPS and 50 ng/mL GM-CSF were added to the culture medium. For the generation of alternatively activated M2 macrophages, 50 ng/mL of macrophage colony-stimulating factor (M-CSF) was added to the culture medium. Parallel setups were differentiated in the presence of 1 µg/mL of the recombinant MERS-CoV spike S1 subunit.

On day 6, macrophage detachment was induced non-enzymatically by incubating the cells in a cold EDTA-HBSS medium. For this, culture medium supernatant was collected on day 6 and stored at −20 °C, and 1 mL of cold HBSS containing 5 mmol/L EDTA was added to the wells. After incubation for 30 min on ice at 4 °C, complete macrophage detachment was achieved by mixing the well content using a pipette. Cell morphology (before detachment) and detachment were evaluated using inverted microscopy. Harvested macrophages were counted on the Accuri C6 flow cytometer (BD Biosciences) after the acquisition of 50 µL of the cell suspension.

### 2.6. Phenotypic Properties of Monocyte-Derived Macrophages

The expression level of cell surface markers was evaluated by flow cytometry after cell labeling with monoclonal antibodies in a two-staining step (indirect labeling). For this, 1 × 10^3^ MDM was incubated in 96-well plates with monoclonal antibodies to the cell surface molecules CD163, MHCII, CD14, CD172a, CD9, CD44, CD18, and CD11a (Table 1) for 15 min at 4 °C on ice. After two washings (3 min at 300× *g*) in cell-staining buffer, secondary FITC-conjugated goat anti-mouse IgG1 and PE-conjugated goat anti-mouse IgG2a were added to the cells for 15 min at 4 °C on ice in the dark. Finally, the cells were washed with staining buffer, resuspended with 100 µL staining buffer, and analyzed by flow cytometry.

### 2.7. Phagocytosis Assay

The phagocytosis activity of camel MDMs was analyzed by flow cytometry after incubating the cells with heat-killed *S. aureus* bacteria (Pansorbin, Calbiochem, Merck, Nottingham, UK) labeled with a labeling kit (FITC, Sigma-Aldrich, St. Louis, MO, USA) according to the manufacturer’s instructions [46]. Camel MDMs (1 × 10^3^ cells in 100 µL RPMI medium) were incubated with *S. aureus*-FITC (20 bacteria/cell) for 30 min at 37 °C and 5% CO_2_. After washing the plate with RPMI medium (300× *g* for 3 min), the cells were resuspended in 100 μL of PBS and analyzed by flow cytometry [47].

### 2.8. Statistical Analyses

Statistical analysis was performed using the software Prism (GraphPad software version 5, GraphPad Software, San Diego, CA, USA). The comparison between the different types of monocyte-derived macrophages was performed using a one-factorial analysis of variance (ANOVA) with Bonferroni’s multiple-comparison test. The results for each analyzed parameter were presented graphically as the means ± standard error of the mean (SEM). The results were considered statistically significant if the *p*-value was less than 0.05.

## 3. Results

### 3.1. Binding of the Recombinant MERS-CoV S1 Protein on the Cell Surface of Camel Mononuclear Cells

**Figure 1:**
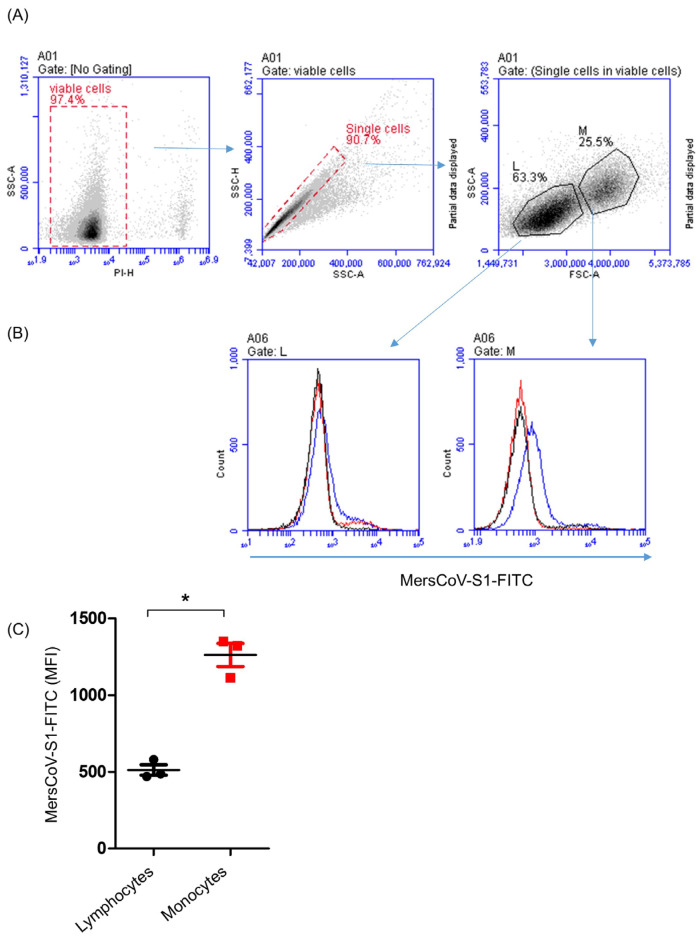
Binding of camel PBMC to the MERS-CoV S1 protein. (**A**) Flow cytometric analysis of camel PBMC labeled with the recombinant MERS-CoV S1 Fc chimera protein in combination with an anti-human IgG-FITC antibody. After excluding dead cells (based on staining with propidium iodide) and cell duplets (based on SSC-A and SSC-H), gates were set on lymphocytes (Ls) and monocytes (Ms) based on their FSC and side-scatter properties. (**B**) The binding of gated lymphocytes and monocytes to the isotype control (red line), human IgG-FITC (black line), and MERS-CoV S1 (blue line) is shown in the form of histograms. (**C**) Mean fluorescence intensity (MFI) of MERS-CoV S1 binding is presented for both cell types (* indicates significant differences; *p* < 0.05; *n* = 3 animals).

Flow cytometric analysis of camel PBMC labeled with the recombinant MERS-CoV S1 Fc chimera protein in combination with an anti-human IgG-FITC antibody revealed selective binding of the recombinant protein to camel monocytes. With a mean fluorescent intensity (MFI) of 1261 ± standard error of the mean (SEM) of 74, camel blood monocytes showed a significantly (*p* < 0.05) higher fluorescence signal than lymphocytes (511 ± 34) (Figure 1A–C).

### 3.2. Generation of Camel Monocyte-Derived Macrophages

**Figure 2:**
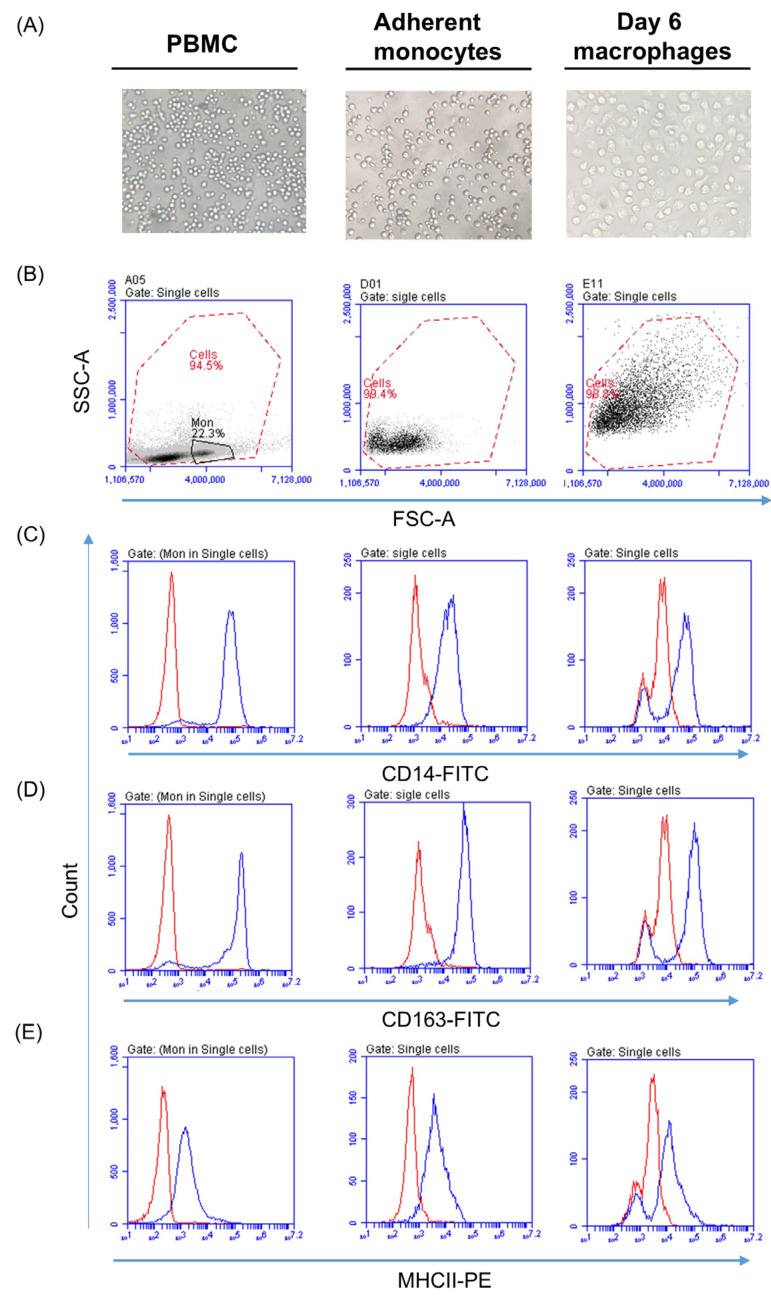
Separated camel PBMC, day 1 adherent monocytes, and day 6 monocyte-derived macrophages (MDMs) were compared in terms of their morphology under a light microscope (**A**), their forward and side-scatter characteristics (**B**), and the expression of the monocytic markers CD14 (**C**), CD163 (**D**), and MHCII (**E**). The expression density of the cell markers is shown as histograms of specific antibody binding (blue line) in comparison to the isotype control (red lines) after gating on the corresponding cell type. The results represent different datasets obtained from different antibody isotype combinations.

Separation of camel PBMCs using density gradient centrifugation yielded a high purity (95.2 ± 1.3%) of PBMCs with high cell vitality (97.3 ± 0.7%) as measured by flow cytometry after incubating the cells with propidium iodide (Figure 1A). Camel monocytes were separated from PBMCs by allowing them to adhere to the wells of cell culture plates and washing out non-adherent lymphocytes. The purity of the separated monocytes was determined based on their positive staining with CD14 antibodies and ranged between 82.6 and 89.1% (85.4 ± 3.7%) of the total cells. Figure 2 shows a comparison between freshly separated camel PBMC, day 1 adherent monocytes, and day 6 monocyte-derived macrophages (MDMs) in terms of their morphology under a light microscope (Figure 2A), their forward and side-scatter characteristics (Figure 2B), and the expression of the monocytic markers CD14 (Figure 2C), CD163 (Figure 2D), and MHCII (Figure 2E). The comparison between monocytes and day 6 MDMs revealed increased MHCII and CD163 expression on the MDMs compared to the monocytes, while the expression density of CD14 was comparable between the monocytes and MDMs (Figure 2C–E).

**Figure 3:**
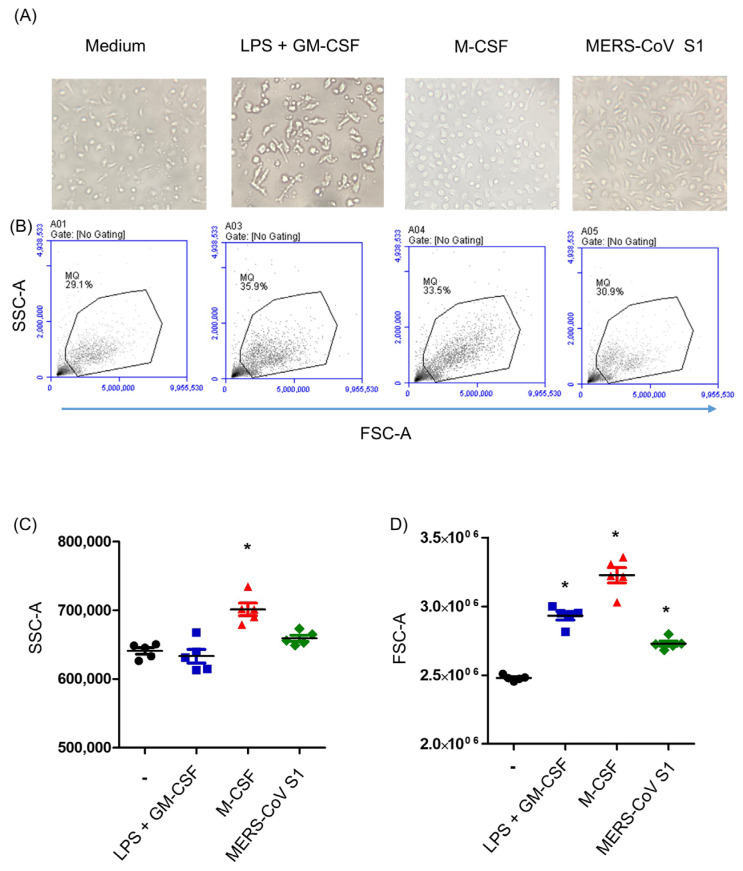
(**A**) Microscopic analysis of the cell morphology of camel MDMs differentiated for 6 days in medium alone (Black), in medium containing LPS and GM-CSF (Blue), in medium containing M-CSF (Red), or in medium containing the recombinant MERS-CoV S1 protein (Green). (**B**) MDM cell size and granularity were measured by flow cytometric analysis of forward-scatter (FSC) and side-scatter (SSC) properties. (**C**) Mean SSC and (**D**) FSC values were calculated and are presented above for the different MDM types (* indicates significant differences; *p* < 0.05; *n* = 5 animals).

Additionally, Figure 3A shows what camel MDM looks like after 6 days of in vitro culture of camel monocytes in medium alone (un-polarized M0 macrophages), medium containing LPS and GM-CSF (LPS-MDM), medium containing M-CSF (M-CSF-MDM), or medium containing the recombinant MERS-CoV S1 protein (MERS-CoV-MDM). It can be seen that the different stimuli induced different changes in macrophage morphology. The cell size and granularity of MDMs were measured based on the forward-scatter (FSC) and side-scatter (SSC) values (Figure 3B). Only M-CSF, an M2 macrophage inducer, resulted in the generation of MDMs with higher granularity than other types of MDMs (*p* < 0.05) (Figure 3C). In comparison to M0 macrophages, the presence of either LPS/GM-CSF, M-CSF, or MERS-CoV S1 protein during differentiation induced a significant increase in MDM cell size, with the highest FSC values for M-CSF-generated MDMs (*p* < 0.05) (Figure 3D).

### 3.3. The Immunophenotype of In Vitro-Generated Camel Monocyte-Derived Macrophages

**Figure 4:**
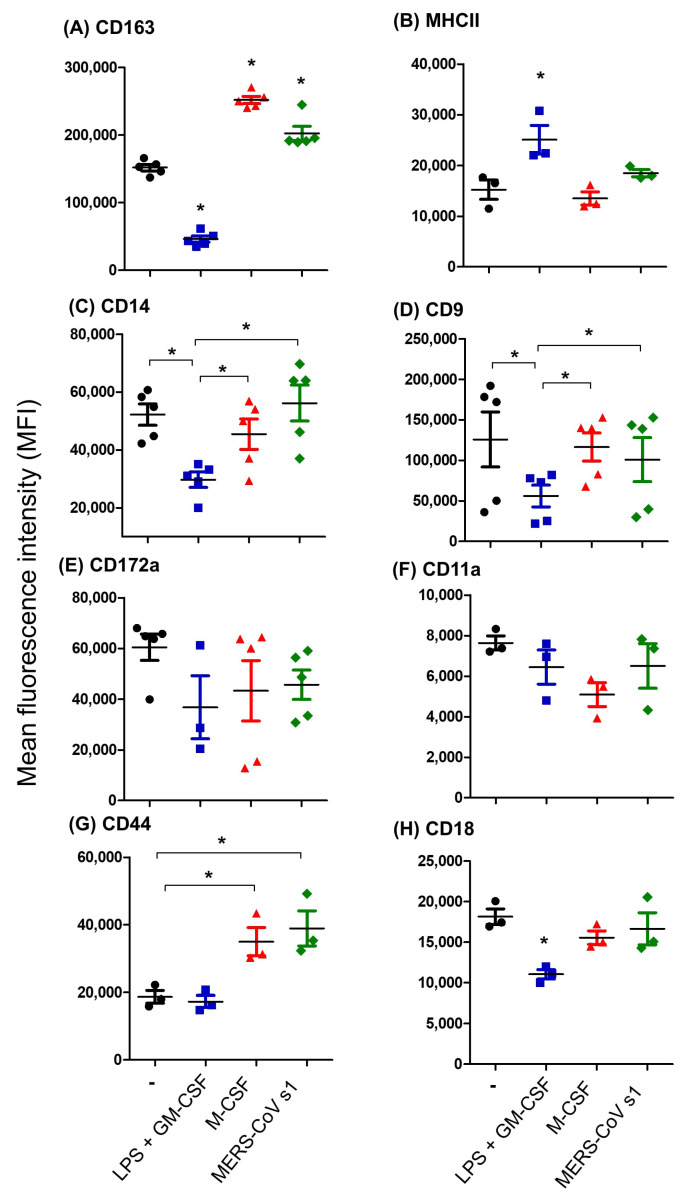
The phenotypic analysis of in vitro-generated camel monocyte-derived macrophages. Camel MDM differentiated for 6 days in medium alone (black), in medium containing LPS and GM-CSF (Blue), in medium containing M-CSF (Red), or in medium containing the recombinant MERS-CoV S1 protein (Green) were labeled with cell marker antibodies and analyzed by flow cytometry. The expression density of CD163 (**A**), MHCII (**B**), CD14 (**C**), CD9 (**D**), CD172a (**E**), CD11a (**F**), CD44 (**G**), and CD18 (**H**) is presented as the mean fluorescence intensity for different types of MDMs (* indicates significant differences; *p* < 0.05).

In comparison to non-polarized MDMs which were generated in culture medium without polarizing stimuli, MDMs generation in the presence of LPS and GM-CSF resulted in a significant decrease (three times lower than the control) in the expression level of CD163 (46,257 ± 4635 mean fluorescence intensity (MFI) vs. 152,027 ± 4843 MFI for non-polarized MDMs) (*p* < 0.05) (Figure 4A), while the expression level of MHCII was significantly (*p* < 0.05) higher on LPS/GM-CSF-polarized MDMs (25,078 ± 2840) than non-polarized MDMs (15,213 ± 1893) (Figure 4B). In contrast to this, the presence of M-CSF significantly (*p* < 0.05) increased the abundance of CD163 (252,067 ± 5387) compared to non-polarized macrophages (152,027 ± 4843 MFI) (Figure 4A). The expression level of MHCII, however, did not differ significantly (*p* > 0.05) between M-CSF-MDMs and non-polarized MDMs (Figure 4B). Similarly to the polarizing effect of M-CSF, the presence of the recombinant MERS-CoV S protein resulted in a significantly higher abundance of surface CD163 (202,636 ± 10,595) without changing the expression level of MHCII (18,474 ± 697) compared to non-polarized MDMs (Figure 4A,B).

The abundance of the LPS receptor CD14 (29,711 ± 2632 vs. 52,195 ± 3680 on control MDM) and the tetraspanin CD9 (56,069 ± 13,390 vs. 125,869 ± 33,943 on control MDM) was significantly lower in camel MDMs differentiated in the presence of LPS and GM-CSF. In contrast to this, MDMs differentiated in the presence of either M-CSF or MERS-CoV S showed a comparable abundance of both CD14 or CD9 molecules, which did not differ significantly (*p* > 0.05) from the expression levels on control MDMs (Figure 4C,D). The expression level of CD172a and CD11a did not differ significantly (*p* > 0.05) between the different groups of MDMs (Figure 4E,F). For the cell surface molecule CD44, a higher expression level was found on MDMs differentiated in the presence of either M-CSF or MERS-CoV S compared to non-polarized or LPS/GM-CSF-polarized MDMs (Figure 4G). The lowest expression of CD18 was found on LPS/GM-CSF-induced MDMs compared to other MDM types (Figure 4H).

### 3.4. Impact of MERS-CoV S1 Spike Protein on the Phagocytosis Activity of Camel MDMs

**Figure 5:**
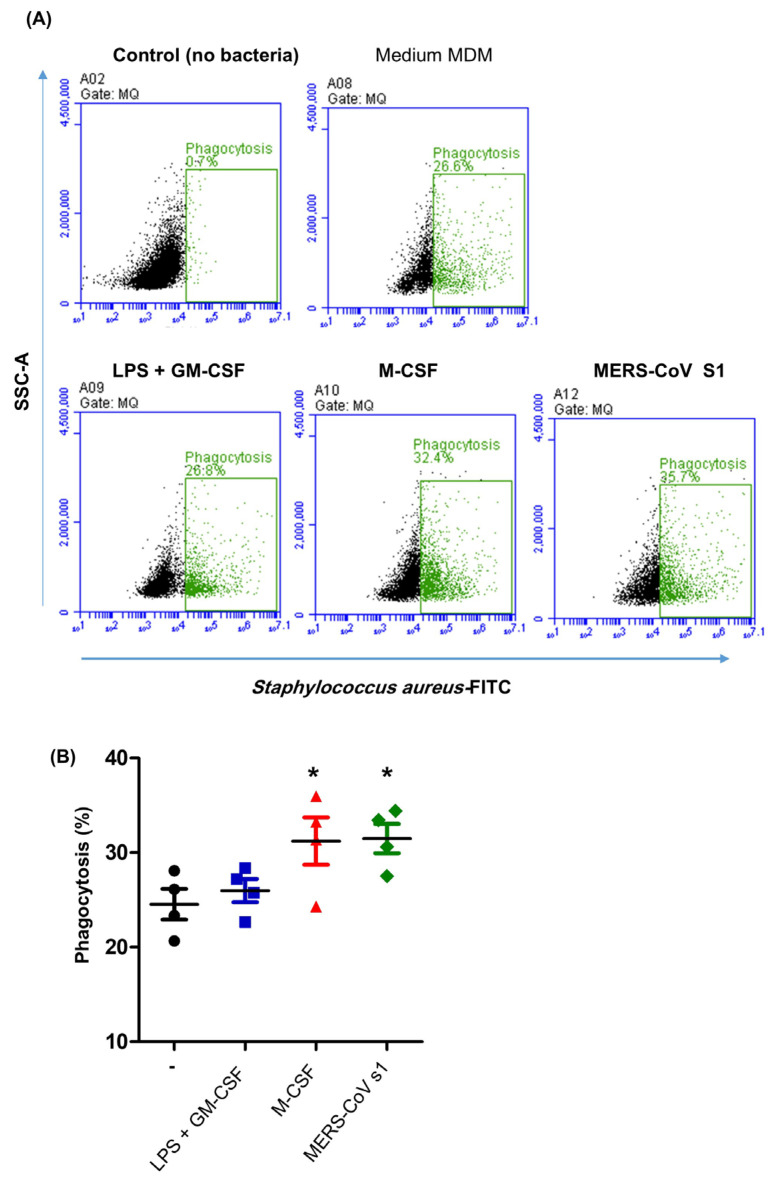
The phagocytosis activity of camel MDMs. Camel MDMs differentiated for 6 days in medium alone (Black), in medium containing LPS and GM-CSF (Blue), in medium containing M-CSF (Red), or in medium containing the recombinant MERS-CoV S1 protein (Green) were incubated with FITC-labeled Staphylococcus aureus. (**A**) Phagocytic cells were identified based on their increased fluorescence in the FITC channel and (**B**) their percentages were calculated and are hereby presented graphically for different types of MDMs (* indicates significant differences; *p* < 0.05; *n* = 4 animals).

The mean percentage of phagocytic cells within the control MDM was 24.5 ± 1.3%. Camel MDMs generated in the presence of LPS and GM-CSF showed comparable (*p* > 0.05) phagocytosis activity, 25.9 ± 1.2%, to the control MDMs. M-CSF (31.2 ± 2.2% of total MDMs) and MERS-CoV S protein (31.4 ± 1.6% of total MDM) induced the differentiation of macrophages with higher phagocytosis activity (Figure 5A,B).

## 4. Discussion

In the present study, the differential binding of the MERS-CoV spike protein to camel mononuclear cells confirmed the selective binding of the virus to camel monocytes rather than lymphocytes and identified monocytes as the target cells for the interaction of MERS-CoV with the camel immune system. Although this binding pattern indicates higher expression of the MERS-CoV receptor on monocytes than lymphocytes, the confirmation of the presence of CD26 (DPP4) on camel monocytes requires the employment of anti-CD26 antibodies, highlighting the need for the development of camel-specific CD26 monoclonal antibodies.

Monocytes are circulating innate immune cells characterized by their plasticity and flexibility, able to differentiate into different functional subtypes depending on the polarizing signals in the tissue microenvironment [34,35]. For humans, mice, and several veterinary species, in vitro differentiation of monocytes into inflammatory or anti-inflammatory macrophage phenotypes has been investigated in several studies, and key polarizing stimuli were characterized [26,27,28,29,30,31,32,33]. Additionally, several alternative MDM polarizing stimuli were identified, including drugs, hormones, neuromodulators, and microbe-derived structures [48,49,50,51,52]. In dromedary camels, the in vitro generation of MDMs has not been investigated so far. The present study generated in vitro-differentiated monocyte-derived macrophages under different polarization stimuli and identified some phenotypic and functional properties. In addition, the impact of the MERS-CoV S protein on the in vitro differentiation of camel monocytes into macrophages was investigated.

Lipopolysaccharide (LPS), granulocyte–macrophage colony-stimulating factor (GM-CSF), interferon-gamma (IFNγ), IL-4, and macrophage colony-stimulating factors (M-CSF) are key polarizing stimuli that have been used for the in vitro generation of polarized pro-inflammatory (classically activated) M1 or anti-inflammatory (alternatively activated) M2 macrophages in several species [26,28,36,38,53,54,55]. In humans and some other species, M1- and M2-polarized macrophages can be identified based on several phenotypic and functional characteristics [38,56]. Antigen presentation receptor major histocompatibility complex (MHC) class II molecules [57,58] and the scavenger receptor CD163 [59] are considered cell surface markers for M1 and M2 macrophages, respectively. In the present study, the reduced expression of the scavenger receptor CD163 on camel MDMs generated in the presence of a combined stimulation of monocytes with LPS and GM-CSF indicated the development of an M1 macrophage phenotype. This was also supported by the increased abundance of MHCII molecules on the LPS/GM-CSF-induced MDMs.

The expression pattern of the cell markers CD163, CD14, CD172a, and CD9 on MDMs generated in the presence of the MERS-CoV S1 protein revealed similarity with M-CSF-induced MDMs, suggesting the potential of the MERS-CoV S1 protein to induce an M2 macrophage phenotype. This was also supported by the higher abundance of the hyaluronic acid receptor CD44 on MDMs differentiated in the presence of the MERS-CoV S1 protein or M-CSF. The upregulation of CD44 has been previously found to be associated with the activation of STAT3 in human monocytes, inducing their differentiation into M2-type macrophages [59]. To ensure that this modulating effect was specific to S protein binding and exclude the role of binding of the FC region to FC receptors in camel monocytes, we included an additional control, showing that fluorochrome-labeled human IgG antibodies did not bind to camel FC receptors in monocytes or lymphocytes (B cells).

Macrophages are specialized mononuclear phagocytic cells that effectively contribute to the ingestion and elimination of bacterial and fungal pathogens [60]. Studies in humans and mice have previously identified several functional differences between M1 and M2 macrophages [61]. Although the development of an M1 phenotype has been found to be associated with pro-inflammatory and antimicrobial functions [61,62,63], some studies have linked polarization toward an M2 phenotype with an enhanced phagocytosis capacity. The anti-inflammatory cytokines IL-4 and IL-10, for instance, stimulate the differentiation of human monocytes toward MDMs with enhanced phagocytosis capability compared to unstimulated cells [64,65,66]. In addition, a positive correlation has been found between phagocytosis activity and CD163 expression in human MDMs [67]. In the present study, M-CSF-induced and MERS-CoV-S protein-induced MDMs showed enhanced phagocytosis activity compared to non-polarized or LPS/GM-CSF-polarized MDMs. This indicates the similarity in phenotype and function between M-CSF-induced and MERS-CoV-S protein-induced MDMs and supports the M2-like phenotype of these MDMs. Alternatively activated M2 macrophages are known for their anti-inflammatory roles, contributing to tissue remodeling and wound healing during the late stages of viral infection [52]. In humans, MERS-CoV has the potential to replicate and establish a productive infection in macrophages, resulting in high pro-inflammatory responses (M1 phenotype) [40], while camelid (llama) alveolar macrophages have been found to be resistant to MERS-CoV replication and not able to induce pro-inflammatory cytokines upon interaction with MERS-CoV [68]. In addition, these llama macrophages were able to effectively capture and degrade viral particles [68]. Whether MERS-CoV-S protein-induced MDMs in camels and MERS-CoV-polarized alveolar macrophages in llamas functionally present the same macrophage subtype is still to be investigated in future studies. The observed enhancement of phagocytic activity in MERS-CoV-S protein-induced camel MDMs supports this theory.

Several studies have reported the capability of viral proteins to stimulate monocytes and induce macrophage polarization with diverse modulatory effects [52,69]. SARS-CoV-2 nucleoprotein induces innate memory in human monocytes [69] and monocyte polarization toward a pro-inflammatory functional subtype [40,41,42] via toll-like receptor (TLR) 4 signaling [70]. Severe cases of highly pathogenic human MERS-CoV infections have been associated with the excessive production of inflammatory cytokines, including IFN-γ, TNF-α, IL-15, and IL-17 [71]. Whether the lack of DPP-4 on monocytes/macrophages in humans could be linked to an uncontrolled pro-inflammatory response, contributing to disease severity, is still to be answered in further studies. The virus may use different receptors to interact with innate myeloid cells in humans (TLRs) and camels (DPP-4), leading to different early inflammatory responses. In addition, an association has been identified between the high human case fatality rate and the downregulation of both Th1 and Th2 immune responses [72]. Given their key role as antigen-presenting cells in the initiation and guidance of the adaptive immune response, the different expression of DPP-4 on monocytes/macrophages may contribute to the difference in disease pathogenesis in humans and camels.

Although the results of the present study provide insight into the role of monocyte and macrophage polarization in immune response to MERS-CoV, the identification of key mechanisms responsible for different disease pathogenesis in humans and camels requires further investigation, especially in relation to the role of other important innate (NK cells, dendritic cells, and γδ T cells) and adaptive (B cells and T cells) immune cells in the interaction with the virus.

## 5. Conclusions

The current study represents the first report on the in vitro generation and polarization of monocyte-derived macrophages (MDMs) in camels and the impact of the MERS-CoV S1 protein on camel MDM phenotype. Although the results suggest a polarizing effect of the MERS-CoV S1 protein on camel MDMs, transforming them into an M2-like phenotype with enhanced phagocytosis activity, these findings need confirmation in functional studies that identify the spectrum of produced M1 or M2 characteristic cytokines or the potential of these macrophages to polarize the T helper cells’ immune response. This highlights the need for the development of camel-specific anti-cytokine antibodies or the identification of cross-reactive antibodies with camel cytokines that can be used for cytokine detection by ELISA or intracellular immunofluorescence. In addition, the clinical relevance of these in vitro findings for disease pathogenesis and camel immune response toward MERS-CoV infection is still to be elucidated. Although the clinical relevance of the role of M2 macrophages in camels’ tolerance to MERS-CoV infection has not yet been proven using adequate in vitro and in vivo studies, the results of the present study strengthen the pivotal role of innate immune cells in the immune response to MERS-CoV. Given the role of macrophages in the biasing of specific T helper cells’ responses to vaccines, we believe that future research may investigate the use of immunomodulatory adjuvants to guide immune response against MERS-CoV vaccines toward the protective T helper immune response type.

## Figures and Tables

**Table 1:** List of antibodies.

Antigen	Antibody Clone	Labeling	Source	Isotype
CD14	CAM36A	-	Kingfisher	Mouse IgG1
CD163	LND68A	-	Kingfisher	Mouse IgG1
MHCII	TH81A5	-	Kingfisher	Mouse IgG2a
CD172a	DH59b	-	Kingfisher	Mouse IgG1
CD9	LT86A	-	Kingfisher	Mouse IgG2a
CD11a	HUH73A	-	Kingfisher	Mouse IgG1
CD44	LT41A	-	Kingfisher	Mouse IgG2a
CD18	6.7	FITC	BD	Mouse IgG2a
Human IgG	poly	FITC	DakoCytomation	Rabbit IgG
Mouse IgG1	poly	FITC	Thermofisher	Goat IgG
Mouse IgG2a	poly	PE	Thermofisher	Goat IgG

MHC: major histocompatibility complex; FITC: fluorescein isothiocyanate; PE: phycoerythrin; and poly: polyclonal.

## Data Availability

The datasets generated during the current study are available from the corresponding author upon reasonable request.

## Abbreviations

The following abbreviations are used in this manuscript:

MERS-CoV	Middle East Respiratory Syndrome Coronavirus
MDMs	Monocyte-derived macrophages
DPP-4	Dipeptidyl peptidase-4
M-CSF	Macrophage colony-stimulating factor
GM-CSF	Granulocyte–macrophage colony-stimulating factor
